# Investigating the Impact of Mechanical Properties and Cell-Collagen Interaction on NIH3T3 Function: A Comparative Study on Different Substrates and Culture Environments

**DOI:** 10.3390/gels9120922

**Published:** 2023-11-22

**Authors:** A Yeon Cho, Hyun Jong Lee

**Affiliations:** Department of Chemical and Biological Engineering, Gachon University, 1342 Seongnam-daero, Seongnam-si 13120, Republic of Korea; cindyjo2007@naver.com

**Keywords:** fibroblasts, matrix stiffness, substrate composition, cell–cell interactions, regenerative medicine

## Abstract

This study investigates the intricate dynamics of matrix stiffness, substrate composition, and cell–cell interactions and elucidates their cumulative effects on fibroblast behavior in different culture contexts. Three primary substrate types were examined: non-coated, collagen-coated, and collagen hydrogel, within both two-dimensional (2D) monolayer and three-dimensional (3D) spheroid cultures. The research provides several key insights. First, 3D spheroid culture, which promotes robust cell–cell interactions, emerges as a critical factor in maintaining fibroblast functionality. Second, substrate stiffness significantly influences results, with the soft collagen hydrogel showing superior support for fibroblast function. Notably, fibroblasts cultured on collagen hydrogel in 2D exhibit comparable functionality to those in 3D, highlighting the importance of substrate mechanical properties. Third, surface composition, as exemplified by collagen coating, showed a limited effect compared to the other factors studied. These findings provide a basis for innovative applications in regenerative medicine, tissue engineering, and drug testing models, and offer valuable insights into harnessing the potential of fibroblasts and advancing biomedical sciences.

## 1. Introduction

The human body functions through an intricate assemblage of diverse tissues that rely on orchestrated behaviors of cellular interactions. Within complex interactions, cell-substrate interactions and cell-to-cell communication are essential to sustaining life processes. The implications of these interactions extend beyond our physiological understanding and influence fields such as tissue engineering and regenerative medicine. The extracellular matrix (ECM) is at the center of a complex interplay regulating various cellular activities, including proliferation, differentiation, motility, and adhesion [1,2]. Understanding the intricacies of cell–ECM interactions enables systematic control of cellular function and facilitates the development of biomaterials to promote favorable cellular behaviors.

The mechanical stiffness of the ECM plays a pivotal role in regulating various aspects of cell–ECM interactions and exerts a complex influence on cellular functions such as survival, growth, and differentiation by modulating cytoskeletal tension [3]. However, the conventional practice of culturing cells on rigid 2D plastic surfaces differs significantly from the physiological conditions found in native tissues. Verma et al. reported that cell proliferation was higher on stiffer substrates, while cells on softer substrates showed cell cycle arrest [4]. Therefore, the effect of the substrate mechanical properties on cellular behavior should be carefully investigated.

At the core of cell–ECM interactions is collagen: a quintessential ECM component. In the living organism, the ECM consists of a dynamic ensemble of molecules, including collagen, fibronectin, laminin, elastin, and proteoglycans [3]. Collagen, in particular, plays a pivotal role as the natural polymer that makes up the skin and serves as a scaffold and culture substrate in tissue regeneration studies. Type I collagen, with its characteristic Gly-X-Y motif, forms the robust collagen fibrils that permeate human tissues, often incorporating proline and 4-hydroxyproline residues that are essential for collagen gelation [5].

Traditional cell culture plates typically lack surface collagen, resulting in limited cell-collagen interactions. To overcome this limitation, collagen-coated surfaces or collagen hydrogel substrates have been developed to facilitate robust cell-collagen interactions with a softer stiffness profile [6]. Shao et al. reported that collagen coating on the titanium surface enhanced the osteogenic differentiation of mesenchymal stem cells by providing an osteoimmune microenvironment [7]. Castro-Abril et al. reported that the stiffness of collagen hydrogel affects the migration of colon cancer cells, and the mechanobiological response of cells can be used as a guide [8]. The introduction of collagen on the surface enhances the bioactivities by linking materials and cells.

In a previous study, we introduced an innovative approach to collagen gelation using riboflavin phosphate (RFP), a biocompatible photosensitizer derived from vitamin B2 [9]. Upon exposure to blue light, RFP is activated, generating free radicals that initiate intermolecular crosslinking, ultimately resulting in collagen hydrogels. These collagen hydrogels form a hydrophilic, biomimetic polymer network that closely mimics the physiological environment and serves as an ideal cell matrix [10]. RFP-mediated collagen crosslinking helps to strongly introduce the collagen matrix to the surface.

Fibroblasts are an appropriate cell to unravel the complex interactions between extracellular matrix (ECM) mechanics, cell-collagen dynamics, and the cell culture environment. These versatile cells, found in the stromal matrix of connective tissues, provide an excellent model for exploring these intricate relationships. Responsible for maintaining tissue integrity and ECM synthesis, fibroblasts are an ideal candidate for studying such interactions [11].

This study compares 2D and 3D cell culture approaches and investigates the interconnected effects of substrate stiffness and collagen presence on cell behavior. We adopt a unified method by utilizing three distinct substrates, comprising conventional culture plates, collagen-coated plates, and collagen hydrogel substrates, to culture fibroblasts in different environments. Analytical techniques, such as AFM analysis, migration studies, MTT assays, and fluorescence measurements, comprehensively assess these interactions. The significance of this study lies in its comprehensive examination of scaffold stiffness and the role of collagen in cell-scaffold interactions, providing valuable insights for biomaterial design, tissue engineering, regenerative medicine, and cancer research [12]. Our goal is to elucidate the intricate interplay of these factors, thereby improving our ability to regulate cellular behavior in various scenarios and fostering a deeper understanding of cellular functions.

## 2. Results and Discussion

### 2.1. Characterization of Collagen Substrate

**Figure 1:**
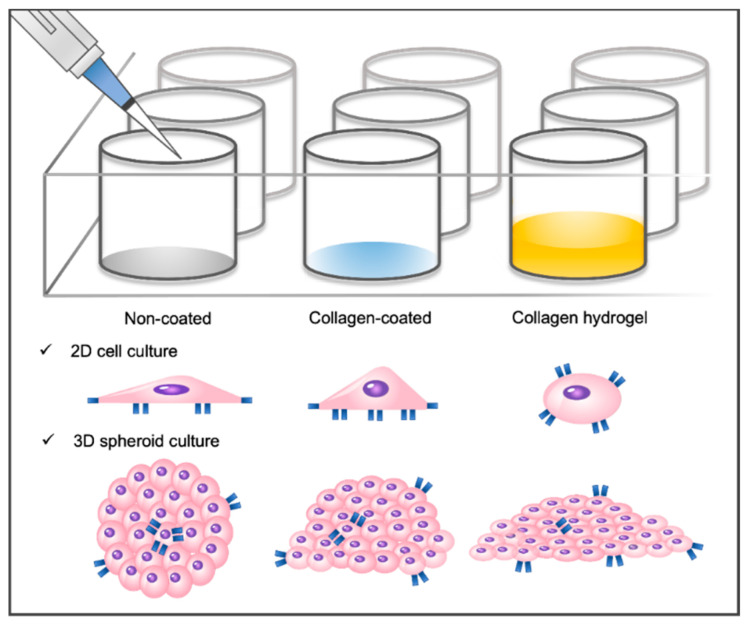
Schematic of the cell culture environment. Three different substrate types are shown: non-coated cell culture plate, collagen-coated cell culture plate, and collagen hydrogel substrate. Two-dimensional monolayer cells and three-dimensional spheroids have been cultured on these substrates.

Three types of cell culture plates were prepared to investigate the combined influence of matrix stiffness and substrate composition on cell–matrix interactions. In the non-coated group, a commercially available cell culture well plate substrate was utilized. The collagen-coated group involved the application of collagen solution to the well plate to enhance cell–collagen interactions while maintaining the original stiffness of the substrate. In the collagen hydrogel group, a collagen hydrogel with a thickness of approximately 0.7 mm was formed in a well plate to create a soft substrate that promotes cell–collagen interactions (Figure 1). A 2D monolayer culture was conducted by seeding NIH3T3 fibroblasts directly onto each prepared substrate. For a 3D spheroid culture, NIH3T3 fibroblast spheroids were formed and then seeded onto the substrate.

The collagen hydrogel was prepared by a crosslinking method using riboflavin phosphate (RFP) as a photosensitizer. This RFP-mediated collagen crosslinking method was used in a previous study and provides a convenient way to adjust the stiffness of the collagen hydrogel by varying the exposure time to blue light [9]. In this study, the blue light exposure time was fixed at 10 min and no further experiments were performed to fine-tune the stiffness of the collagen hydrogel. However, the exposure time can be easily modified depending on the desired cell type and specific research objectives.

**Figure 2:**
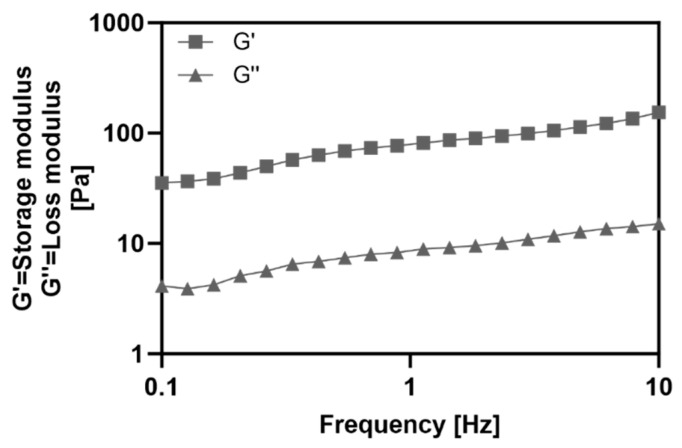
Frequency dependent storage (G′) and loss (G″) moduli of the collagen hydrogel substrate as determined by the rheometer.

To evaluate the properties of the collagen hydrogel, the storage modulus (G′) and loss modulus (G″) were measured using a rheometer (Figure 2). Frequency sweep rheology determines the relationship between G′ and G″ of a material at different frequencies, providing a rheological method to analyze viscoelastic properties and material state [13]. Throughout the frequency range, G′ showed higher values than G″, indicating that the collagen hydrogel exhibited the behavior of gel.

**Figure 3:**
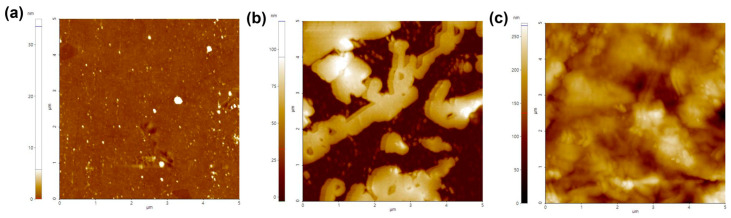
Atomic force microscope (AFM) images of substrate surfaces. (**a**) Non-coated, (**b**) collagen-coated, and (**c**) collagen hydrogel substrates.

Based on the successful formation of the collagen hydrogel, a comparative analysis was conducted on the characteristics of the three substrates. First, we measured the average roughness of each substrate (Figure 3). The results obtained by calculating the standard deviation of the root mean square roughness (Rq) of the matrix surface indicated an increased surface roughness for the collagen-coated (32.78 ± 5.47) nm and the collagen hydrogel substrate (156.20 ± 5.64) nm compared to the non-coated substrate (0.14 ± 1.00) nm. The higher standard deviation for the non-coated substrate was attributed to surface contamination. The collagen hydrogel substrate had approximately five times the roughness of the collagen-coated substrate. It was evident that the non-coated surfaces maintained a flat and smooth appearance while the collagen-coated surfaces exhibited increased roughness due to the collagen coating. Although the collagen hydrogel showed a relatively small difference from the collagen-coated, it had the highest roughness of all the substrates, with all substrate roughness measurements falling in the nanometer range.

Cell–surface interactions play a critical role in cell attachment, proliferation, and tissue regeneration, and the surface topography of biomaterials significantly influences cell behavior and biocompatibility [14]. Typically, surface roughness determines the surface area available for cell attachment and growth, thus influencing initial cell adhesion and interaction with the extracellular matrix. While the measured substrate roughness varied, they were all in the nanometer range and were small compared to the cell size. Therefore, we concluded that the roughness of the three substrates was not a significant factor in inducing substantial changes in cell behavior.

**Figure 4:**
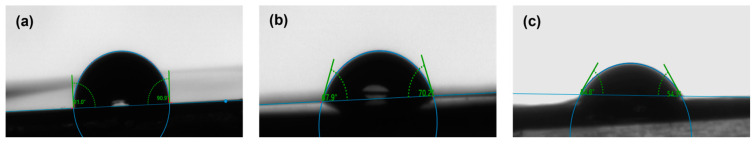
Contact angle analysis of substrates. (**a**) Non-coated, (**b**) collagen-coated, and (**c**) collagen hydrogel substrates. Images of water droplets on the surface.

Another factor that can influence cell behavior is the wettability of the substrate surface. Substrate wettability is a critical factor in cell adhesion and determines cell spreading, migration, and proliferation [15]. Measurement of the contact angle by water contact angle analysis revealed contact angles of 90.69 ± 0.64° for the non-coated substrate, 71.53 ± 1.99° for the collagen-coated substrate, and 56.87 ± 2.33° for the collagen hydrogel substrate (Figure 4). Coating the surface with collagen reduced the contact angle due to the hydrophilicity of collagen. In addition, the contact angle of the collagen hydrogel, which contains water in the matrix, decreased even more compared to the collagen coating.

In general, cells adhere most effectively to surfaces with contact angles between 40 and 70°, and for fibroblast cells in particular, the highest adhesion strength is achieved when the contact angle is between 60 and 80° [15,16]. Overall, all surfaces provided contact angles within a range suitable for fibroblast cell attachment. Notably, the collagen-coated substrate theoretically provided the most favorable contact angle. By culturing cells on substrates with different contact angles, we established conditions ideal for examining cell–matrix interactions while considering both stiffness and wettability.

Three substrates, non-coated, collagen-coated, and collagen hydrogel, were characterized for their effect on cell–matrix interactions. The collagen hydrogel, prepared by riboflavin phosphate-mediated crosslinking, exhibited viscoelastic properties. Comparative analysis revealed increased surface roughness for coated and hydrogel substrates, with the latter having the highest roughness. Surface wettability varied but provided suitable conditions for fibroblast attachment. Furthermore, the chemical composition of the collagen-coated and collagen hydrogel substrates was the same (Figure S1). These characterizations provide the basis for understanding the influence of the substrate on cell behavior in subsequent analyses.

### 2.2. Spheroid Behavior and Migration

**Figure 5:**
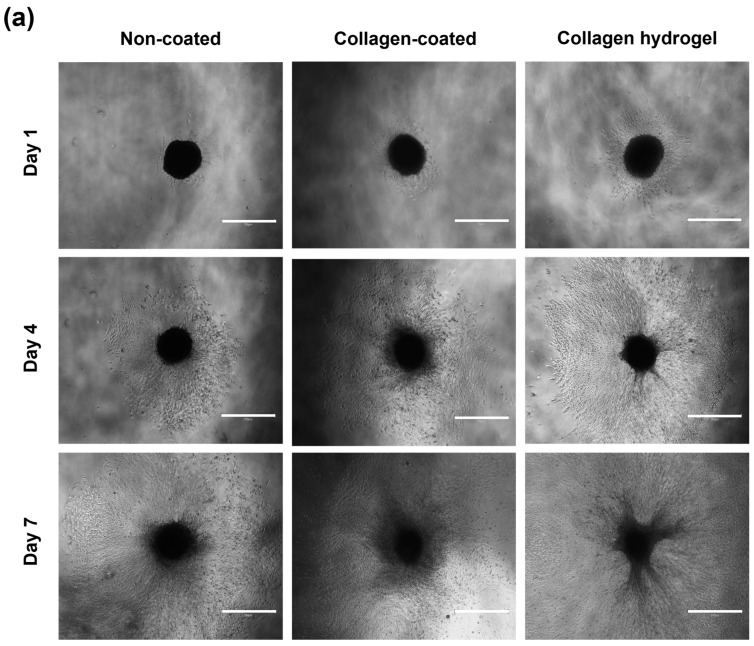

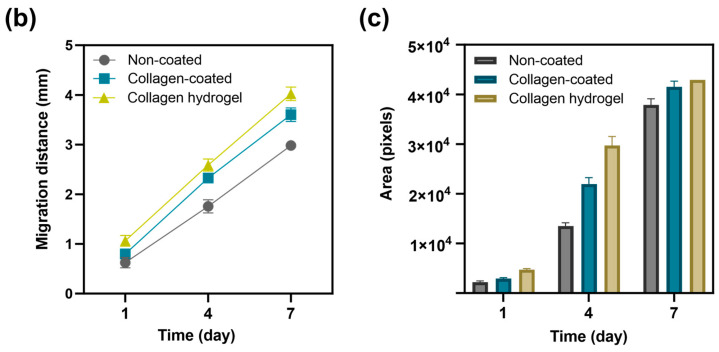
Analysis of spheroid migration. (**a**) Images of spheroid migration on non-coated, collagen-coated and collagen hydrogel substrates on days 1, 4, and 7. Scale bars: 750 μm. (**b**) Migration distance of spheroid is an average of horizontal, vertical, and diagonal. (**c**) Migration area was quantified using ImageJ software (version 1.52a). Data are based on triplicate measurements (n = 3).

A round bottom plate was coated with a coating solution and 2 × 10^3^ NIH3T3 cells were seeded to form 3D spheroids. The size of the resulting spheroids and their migration over time were measured (Figure 5). The initial diameter of the spheroids was 510 ± 44 μm. After the spheroids were transferred to each substrate, the average diameter and area of the spheroids were measured on days 1, 4, and 7. The average diameters and areas for spheroid migration on the non-coated, collagen-coated, and collagen hydrogel substrates on days 1, 4, and 7 are shown in Table 1.

Diameter represents the distance that cells migrated from the center of the spheroid, while area was quantified using ImageJ software (version 1.52a) to measure the area where cells were attached in the images. In all groups, there was an increasing trend in the distance and area of cell migration from the center of the spheroid to the substrate surface over time. The spheroids on collagen hydrogel substrates showed the most extensive migration, followed by collagen-coated and non-coated substrates, which showed a gradual decrease in migration over time.

The surfaces of collagen-coated and collagen hydrogel substrates provide abundant cell–collagen interactions, suggesting that cell migration may be more active compared to the non-coated substrate. Collagen is a major protein component of the extracellular matrix (ECM) and plays a critical role in cell attachment and proliferation by regulating cytoskeletal rearrangement and signal transduction [17]. In contrast, cells on the non-coated substrate were confined to a smaller area over time, reflecting slower cell migration due to reduced cell–matrix interaction on an untreated surface.

The observed differences in cell migration between the different substrates prompt a discussion on the interrelated influence of surface properties on cell behavior. The enhanced migration on collagen-coated and collagen hydrogel substrates, attributed to enriched cell–collagen interactions, is consistent with the well-established role of collagen in facilitating cell attachment and proliferation through cytoskeletal rearrangement and signal transduction mechanisms [17]. In contrast, the slower migration on the non-coated substrate highlights the importance of surface treatment in promoting effective cell–matrix interactions.

Notably, the comparison between collagen-coated and collagen hydrogel substrates revealed a higher migration rate on the hydrogel despite similar collagen-based surfaces. This intriguing finding suggests that factors beyond surface composition, such as surface wettability and stiffness, contribute to the observed differences in cell behavior. While performing dedicated experiments to individually assess the effects of surface wettability and stiffness was challenging due to their interrelated nature, the collective effect of these surface properties on cell migration is evident from the observed patterns.

The significant influence of surface material composition, wettability, and stiffness collectively highlights the need for a holistic understanding of substrate properties in guiding cell behavior. This nuanced perspective is critical for the design of biomaterials in various biomedical applications, ranging from regenerative medicine to tissue engineering, where precise control of cell migration and interactions is paramount.

### 2.3. Evaluation of Cell Proliferation, Morphological Changes, and Extracellular Matrix Secretion

**Figure 6:**
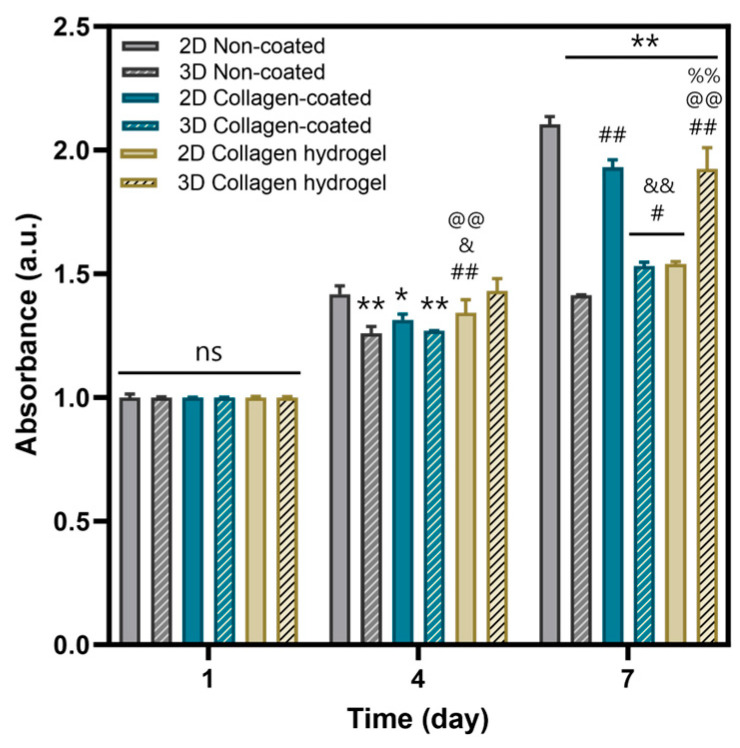
Cell viability of non-coated, collagen-coated, collagen hydrogel substrates of 2D and 3D by MTT assay in day 1, 4, 7. Bars represent the mean ± SD (n = 3). Significant differences are denoted 0.01 < *p* < 0.05 (*, #, &), *p* < 0.01 (**, ##, &&, @@, %%). The symbols * indicate a difference from the 2D non-coated, # from the 3D non-coated, & from the 2D collagen-coated, @ from the 3D collagen-coated, and % from the 2D collagen hydrogel substrates.

MTT assays were performed to evaluate cell proliferation on the three types of substrates (Figure 6). In addition, to investigate the effects of cell–cell interaction, experiments were divided into two categories: 2D cell culture and 3D spheroid culture. Considering 2D and 3D culture conditions, cell proliferation on non-coated and collagen-coated substrates was more active in 2D culture than in 3D spheroid culture. In contrast, on collagen hydrogel substrates, 3D spheroid proliferation exceeded that of 2D culture.

When comparing proliferation rates between 2D and 3D spheroid cultures, 2D cultures generally showed more active proliferation. In 2D culture, cells are seeded as single cells, providing enough space to ensure low cell density upon cell attachment to the substrate. In 3D spheroid culture, cells are densely packed and proliferation occurs primarily as cells in the outermost layers of the spheroid migrate along the substrate. Thus, a proliferation of 2D cultured cells was more active than 3D spheroid culture on both non-coated and collagen-coated substrates. However, collagen hydrogel substrates showed the opposite result. Fibroblasts in 2D culture on collagen hydrogel substrates did not show robust proliferation compared to other substrates, but 3D spheroids on these substrates showed more active proliferation. As suggested by the migration results in Figure 5, it can be inferred that the cells forming the spheroids on collagen hydrogel substrates migrated smoothly, resulting in more active proliferation than on other substrates.

**Figure 7:**
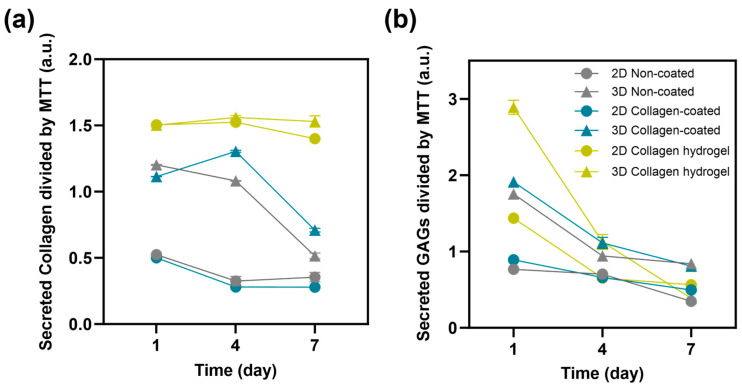
Protein release of 2D and 3D non-coated, collagen-coated and collagen hydrogel substrates using (**a**) collagen and (**b**) glycosaminoglycan assay on days 1, 4, 7 (n = 3).

In addition to cell proliferation, the secretion of collagen and glycosaminoglycans (GAGs) from the cells was measured to assess their functional performance (Figure 7). Fibroblasts are cells that play an important role in the composition of the extracellular environment, with collagen and GAGs as their major secreted factors [18,19,20]. To normalize the total collagen and GAGs secretion to the function of each cell, it was divided by the measured MTT values. Collagen secretion was most active in cells cultured on collagen hydrogel substrates, and there was little difference between 2D and 3D cultures. Notably, collagen secretion remained consistently high from day 1 to day 7 without a decrease. For the other substrates, non-coated and collagen-coated, cell seeding conditions rather than substrate type had a greater influence on collagen secretion. In all cases, 3D spheroid cultures showed higher collagen secretion than 2D cultures. However, in 3D culture conditions, collagen secretion was maintained until day 4 and then decreased significantly by day 7. In 2D culture conditions, there was a steady, low level of collagen secretion from day 1 to day 7.

The secretion of GAGs showed a decreasing trend over time in all groups, with almost indistinguishable secretion levels on day 7 in all groups. On day 1, cells cultured in 3D spheroids on collagen hydrogel substrates showed significantly higher secretion of GAGs. Subsequently, cells cultured in 3D spheroids on collagen-coated and non-coated substrates also showed higher secretion of GAGs. Cells cultured on collagen hydrogel in 2D culture conditions showed a GAGs secretion level similar to that of 3D spheroids cultured on non-coated substrates and higher than the other 2D cultures.

Except for 2D cultures on collagen hydrogel substrates, all groups showed higher collagen and GAG secretion in 3D spheroid cultures compared to 2D cultures. This suggests that cell–cell interactions play an important role in influencing fibroblast function in 3D spheroid cultures. The intercellular space in spheroids, where proteins such as fibronectin, collagen, and GAGs are secreted and accumulated, is known to be very active in 3D culture conditions [21]. When fibroblasts were cultured on collagen hydrogel substrates, cells in the 2D culture conditions exhibited levels of function that were higher than or similar to those of 3D spheroids on other substrates. This suggests that while cell–cell interaction is critical, adequate cell–matrix interaction can sufficiently maintain cell functionality.

### 2.4. Immunofluorescence Analysis

**Figure 8:**
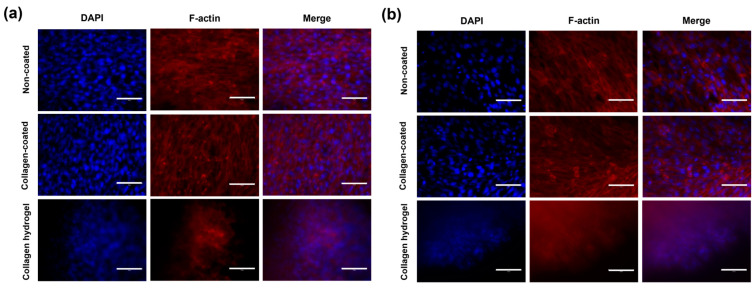
F-actin expression in immunofluorescence staining images in (**a**) 2D cell culture and (**b**) 3D spheroid culture on day 7. The blue fluorescence in the nucleus shows DAPI staining, and the red fluorescence represents the location of F-actin. Scale bars: 75 μm.

Immunofluorescence analysis was used to evaluate cell morphology and expression to better understand the effects of cell–cell and cell–matrix interactions on cells. Cell morphology was visualized using phalloidin, which binds selectively to F-actin filaments (Figure 8). Overall, a fibrous, linear structure was observed in all groups. In 2D cell culture conditions on non-coated and collagen-coated substrates, cells spread over a wide area, with F-actin appearing sharply linear. However, on collagen hydrogel substrates, cells were densely packed even in 2D culture conditions. In addition, F-actin expression was relatively low.

The central role of F-actin in shaping the cellular cytoskeleton and influencing cell structure, adhesion, and functions such as cell movement and muscle contraction is well established [22]. Recent studies indicate that matrix stiffness plays a critical role in regulating the reorganization and morphological distribution of the cellular cytoskeleton, with a positive correlation observed between matrix stiffness and F-actin expression [23,24]. In the context of 2D culture on collagen hydrogel, which is characterized by a dense cell structure, an environment was created that promotes enhanced cell–cell interactions, providing valuable insights into the influence of matrix stiffness on cellular dynamics.

When staining by 3D spheroid culture, it was difficult to observe the central part of the spheroid due to the high cell density, so the focus was on the area extending from the center of the spheroid due to migration. On non-coated substrates, where cell migration was not active, a relatively high density of F-actin was observed. However, unlike what was seen on collagen hydrogel substrates, the cells were not clustered but were individually attached to the substrate, maintaining their spacing. Collagen-coated substrates showed a relatively lower cell density compared to non-coated substrates, but the overall results were similar. On collagen hydrogel substrates, in both 2D and 3D cultures, F-actin images were faint, and the cellular cytoskeleton was not clearly visible, with an overall tendency for cells to cluster.

In 3D spheroid culture, similar to 2D culture, it was observed that cells cultured on stiff substrates, such as non-coated and collagen-coated substrates, promoted fibrotic transformation mechanisms. This is characteristic of fibroblasts and the pressure exerted on the cells by the stiffness of the matrix affects the cells, increasing contractility and decreasing cell mobility [11]. When comparing non-coated and collagen-coated substrates, it was evident that the cell cytoskeleton was influenced by both substrate stiffness and composition but showed a greater dependence on matrix stiffness.

**Figure 9:**
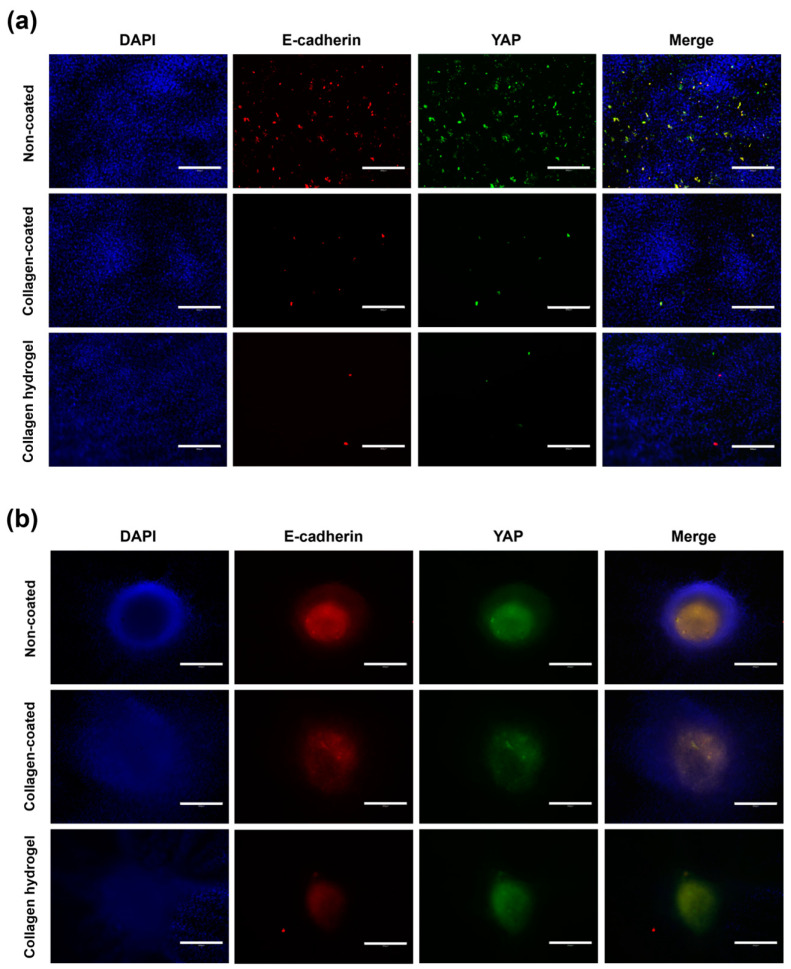
E-cadherin and YAP expression in immunofluorescence staining images in (**a**) 2D cell culture and (**b**) 3D spheroid culture on day 7. The blue fluorescence in the nucleus shows DAPI staining, the red fluorescence represents E-cadherin, and the green fluorescence represents the location of YAP. Scale bars: 300 μm.

To evaluate the influence of matrix stiffness and cell–cell interaction, the expression levels of Yes-associated protein (YAP) and E-cadherin were observed by immunostaining on day 7 (Figure 9). In 2D cell culture, cells on non-coated substrates exhibited a general distribution of YAP and E-cadherin staining and showed the highest relative fluorescence intensity. In contrast, collagen-coated and collagen hydrogel substrates showed relatively narrow areas of fluorescence with significantly lower fluorescence intensity compared to non-coated substrates. Collagen-coated substrates showed a more extensive presence of YAP and E-cadherin compared to collagen hydrogel substrates, although the difference in fluorescence intensity of YAP was not observed, E-cadherin fluorescence intensity was slightly higher in collagen-coated substrates than in collagen hydrogel substrates.

YAP is a protein that senses various physical cues, including matrix stiffness and mechanical forces associated with cell structure and the cytoskeleton. It translates these cues into cell-specific transcriptional programs that induce gene expression [25]. Cells induced by a stiff ECM require YAP function, whereas cells associated with a soft ECM require inactivation of YAP function, thereby suppressing YAP activity [26]. E-cadherin is a core component of adherens junctions, which are essential for cell adhesion and maintenance of epithelial phenotypes. It anchors cells and promotes cell–cell interactions by physically restraining cell movement [27]. In addition to promoting cell adhesion, E-cadherin initiates signaling that regulates cell shape, movement, proliferation, differentiation, and survival [28].

In 2D cell culture, the observed increase in YAP activity was likely due to individual cells experiencing increased stiffness due to the rigid substrate. This is because stiff substrates transmit mechanical forces directly to the nucleus, resulting in nuclear flattening and increased YAP nuclear translocation [26]. Since the non-coated substrate had no collagen treatment, cell–cell interactions were most likely due to the lack of collagen, which is why the epithelial marker E-cadherin was also expressed at high levels. Collagen hydrogel substrates had very low stiffness due to their soft matrix properties, which exerted minimal pressure on the cells, resulting in inhibition of YAP activity. YAP signaling was found to be responsive to mechanical transitions in cell shape, cytoskeletal integrity, and mechanical forces throughout the cell or tissue, and it is known that an increase in F-actin cytoskeletal assembly leads to increased nuclear YAP [27]. This is consistent with the immunofluorescence results showing increased YAP activity on non-coated and collagen-coated substrates where the F-actin cytoskeleton was prominently displayed. Furthermore, although collagen matrix-cell interaction was active, cell–cell interaction was very weak, resulting in the suppression of E-cadherin expression. Collagen-coated substrates, which were similar to non-coated substrates in terms of substrate stiffness, had little effect on 2D cells, suggesting that the expression of YAP and E-cadherin in 2D cell culture is more associated with the presence of collagen.

In 3D spheroid culture, the prominent intensity of E-cadherin and YAP was observed in the center of the spheroid. This expression was primarily attributed to cell–cell interactions within the spheroid rather than cell–matrix interactions. As a result, differences in the distribution and intensity of E-cadherin and YAP between groups were not as pronounced as in the previous 2D culture. In the 2D culture, E-cadherin and YAP expression was highly localized and showed strong intensity, while within the spheroids, the expression was more evenly distributed throughout the spheroid rather than restricted to specific areas. In particular, the distribution of E-cadherin and YAP was concentrated in the inner regions of the spheroid rather than in the outer regions. Notably, the staining area of spheroids on collagen hydrogel substrates was narrower compared to the other two substrates.

In general, cells cultured at low density show reduced expression of the epithelial marker E-cadherin [29]. Consequently, in 3D culture, strong E-cadherin expression was centered around the spheroid core, which had a high cell density and was rarely observed in 2D culture. YAP activity was also inhibited except in the spheroid center, where the low stiffness of the collagen and extensive cell movement resulted in minimal pressure, making it the most biologically relevant collagen substrate environment for 3D spheroid culture. The inhibitory effect of low mechanical stress on YAP activity has been well documented [30]. Thus, it was confirmed that YAP and E-cadherin expressions are primarily influenced by cell density and cell–cell interactions, with secondary effects from cell–matrix interactions.

This research provided insight into the key factors influencing fibroblast behavior under 2D and 3D culture conditions and the influence of substrate type. The most significant factor identified was cell–cell interaction in 3D spheroid culture, which minimized the effect of substrate and maintained fibroblast functionality. Substrate stiffness had a significant effect, with the soft collagen hydrogel substrate significantly improving fibroblast function compared to stiff substrates. Of particular significance, fibroblasts cultured on collagen hydrogel substrates in a 2D environment exhibit similar cell functionality as those cultured in 3D. Surface composition had the least effect, with collagen-coated surfaces showing no significant differences from non-coated surfaces in all experiments. The research elucidates the impact of cell–cell and cell–matrix interactions, as well as stiffness, on cellular behavior. This has significant future implications in regenerative medicine, including tissue engineering and drug screening, where cellular approaches play a crucial role.

## 3. Conclusions

In this study, we investigated the intricate interplay of matrix stiffness, substrate composition, and cell–cell interactions and elucidated their collective influence on fibroblast behavior in different culture environments. The dominant variable that surfaced was the significant influence of 3D spheroid culture, in which robust cell–cell interactions were prioritized to maintain fibroblast function. These findings emphasize the importance of promoting robust cell–cell junctions in microenvironments, which indicates potential for their application in regenerative medicine. In addition, substrate stiffness emerged as a critical determinant of fibroblast responses, with the soft collagen hydrogel substrate significantly enhancing fibroblast function. Even in a 2D culture, fibroblasts on collagen hydrogel substrates retained similar functionality as their 3D counterparts. This emphasizes the fundamental importance of substrate mechanical properties in directing cellular behavior and implies that soft substrates may be advantageous in enhancing cell function. Finally, the results indicate that surface composition, as demonstrated by collagen coating, had a minor impact compared to other investigated factors. The research highlights that the presence of collagen coating did not trigger noteworthy deviations from non-coated surfaces. This confirms the superiority of factors such as stiffness and cell–cell interactions in shaping fibroblast behavior. These findings have significant implications for regenerative medicine, tissue engineering, and drug testing models. They provide the basis for innovative strategies to harness the potential of fibroblasts in various applications and paving the way for advances in biomedical sciences. Future research on the molecular mechanisms and diverse cell types involved will enhance our comprehension and capacity.

## 4. Materials and Methods

### 4.1. Materials

Unless otherwise stated, all chemicals and solvents were used as supplied by the manufacturer. Dulbecco’s modified Eagle’s medium (DMEM), fetal bovine serum (FBS), phosphate-buffered saline (PBS, pH 7.4), Dulbecco’s phosphate-buffered saline (DPBS), and penicillin-streptomycin were purchased from WelGene, Inc. (Daegu, Republic of Korea). Bovine collagen I and 3-(4,5-dimethylthiazol-2-yl)-2,5-diphenyltetrazolium bromide (MTT) were purchased from Invitrogen (Carlsbad, CA, USA). BIOFLOAT™ FLEX coating solution was purchased from faCellitate (Mannheim, Germany). Triton X-100, riboflavin phosphate (RFP), 4′,6-diamidine-2′-phenylindole dihydrochloride (DAPI), and bovine serum albumin (BSA) were purchased from Sigma-Aldrich (St. Louis, MO, USA). Sircol™ soluble collagen assay kit and Blyscan™ sulfated GAGs assay kit were purchased from Biocolor (Carrickfergus, UK). The F-actin staining kit was purchased from Abcam (Cambridge, UK). Paraformaldehyde, Alexa Fluor^®^ 594 anti-human CD324 (E-cadherin) antibody (clone: 67A4), rabbit yes-associated protein (YAP) polyclonal antibody, Alexa Fluor™ 488 goat anti-rabbit IgG (H + L), and Rhodamine Red™-X goat anti-mouse IgG (H + L) were purchased from Thermo Fisher Scientific (Waltham, MA, USA).

### 4.2. Fabrication and Characterization of Collagen Substrates

The non-coated surface was provided by commercially available cell culture well plates. The collagen-coated substrate was prepared by coating the well plates with collagen solution. A 200 μL solution of diluted collagen (0.01% *w*/*v* in DI water) was added to the well and incubated overnight before removing the solution. The collagen hydrogel substrate was prepared by RFP-mediated photocrosslinking. The pH of the collagen solution was adjusted to 7 by mixing with distilled water, sodium hydroxide, and 10× phosphate-buffered saline. RFP (0.01% *w*/*v* in PBS) was then added to the neutralized collagen solution. Next, 200 μL of collagen hydrogel precursor solution was added to the well and exposed to blue light for 10 min.

The mechanical properties of the collagen hydrogel substrate were analyzed using a rheometer (MCR92, Anton Paar, Graz, Austria). A total of 200 μL of collagen solution (0.01% *v*/*v*) containing RFP was applied to the rheometer plate and exposed to blue light for 5 min. Analysis was performed using an 8 mm parallel plate (PP08) with fixed parameters of storage modulus (G′), loss modulus (G″), and shear strain set to 1%. A frequency sweep was performed over a range of 0.1 Hz to 10 Hz and measurements were performed at a constant temperature of 37 °C.

The surface roughness was measured using an atomic force microscope (Park Systems Corp., Suwon, Republic of Korea). In the case of collagen-coated plates, the process was initiated by applying a dilute collagen solution (0.01% *v*/*v* in distilled water) to a glass slide. This solution was washed with distilled water for 24 h, then removed and dried. For collagen hydrogel substrate sampling, a neutralizing solution diluted with distilled water and a collagen solution (0.01% *v*/*v*) containing RFP was applied to a glass slide. Another glass slide was carefully placed on this assembly and exposed to blue light for 10 min. As a control, we introduced non-coated flat glass slides without any treatment. All imaging processes were performed in non-contact mode and at room temperature to ensure the accuracy of our characterizations.

The wettability was measured with a contact angle meter (DSA100, KRUSS, Hamburg, Germany). The drop phase was water, and the drop type was set to sessile drop. The untreated flat plastic plate (uncoated) was used as a control group for comparison.

### 4.3. Cell Culture and Spheroid Formation

NIH3T3 cells were obtained from the Korean Cell Line Bank (Seoul, Republic of Korea) and cultured in an incubator maintained at 37 °C with 5% CO_2_ and 95% air. The cell culture medium used was DMEM supplemented with 1% *v*/*v* penicillin and 10% *v*/*v* FBS. For two-dimensional (2D) cell culture, cells were grown to 90% confluence on a T-75 plate. The experiment was then initiated by seeding 2 × 10^3^ NIH3T3 cells into a 48-well plate. For three-dimensional (3D) spheroid culture, 100 μL of a BIOFLOAT™ FLEX coating solution was added to an untreated round-bottom 96-well plate and incubated for 3 min. After the coating solution was removed and allowed to air dry at room temperature for 30 min, 2 × 10^3^ NIH3T3 cells were seeded onto the coated well plate. After aggregation of the cells in the center, facilitated by a shaker, and incubation for 24 h, the resulting spheroids were transferred to a 48-well plate for the experiment.

### 4.4. Evaluation of Cell Proliferation, Morphological Changes, and Extracellular Matrix Secretion

To evaluate cell proliferation and viability under different culture conditions, 2 × 10^3^ NIH3T3 cells were seeded for 2D monolayer and 3D spheroids. On days 1, 4, and 7, 100 μL of MTT solution (6 mg/mL) was added and then incubated the cells at 37 °C for 2 h. After removing the solution, formazan crystal was dissolved in 650 μL of DMSO and transferred 200 μL of this solution to 96-well plates. The spectrophotometer (Microplate Spectrophotometer, Epoch.Co, Tokyo, Japan) was used to measure the absorbance at 540 nm.

We initiated our investigation by cultivating 3D spheroids, which were formed by seeding cells on days 1, 4, and 7. To comprehensively assess their morphological evolution, we meticulously measured the horizontal, vertical, and diagonal dimensions of these spheroids. Subsequently, we calculated their mean values to provide an overall description. In addition, the migratory area of the spheroids was quantified using ImageJ software (version 1.52a).

Secreted collagen was quantified using a Sircol™ soluble collagen assay kit. Media collected on days 1, 4, and 7 were mixed with 1 mL of collagen assay solution in tubes and shaken on an orbital shaker for 30 min. The mixture was then centrifuged for 10 min, and the supernatant discarded. Ice cold acid salt (750 μL) was added, centrifuged for 10 min, and the supernatant discarded. Alkaline reagent (250 μL) was added to the pellet and vortexed. The resulting solution was transferred to a 96-well plate, and the absorbance was measured at 556 nm using a spectrophotometer.

Secreted GAGs were quantified using a Blyscan™ sulfated GAGs assay kit. Media collected on days 1, 4, and 7 were mixed with 1 mL of GAGs in tubes and shaken on an orbital shaker for 30 min. The mixture was then centrifuged for 10 min, and the supernatant was discarded. A dissociation reagent (500 μL) was added to the pellet, followed by vortexing and further centrifugation for 5 min. The supernatant was collected and transferred to a 96-well plate, and the absorbance was measured at 656 nm using a spectrophotometer.

### 4.5. Immunocytochemistry for Protein Expression

On day 7 of the cell culture, the cells were fixed with a 4% *w*/*v* paraformaldehyde solution in PBS for 20 min, followed by two thorough PBS washes. F-actin staining was performed using Triton red fluorescent phalloidin from the F-actin staining kit, applied for one hour, then subjected to two PBS washes. DAPI staining was introduced for 15 min. Immunocytochemistry was conducted to assess the expression of YAP and E-cadherin, aiming to discern the influence of substrate characteristics and cell–cell interactions across distinct cell culture environments. To minimize non-specific antibody binding, a bovine serum albumin blocking buffer (5% *w*/*v* in PBS) was used, diluted with PBS and applied for 20 min. After two subsequent PBS washes, the primary antibody was incubated with BSA (1% *w*/*v* in PBS) for two hours. The primary antibodies used in this study were Alexa Fluor^®^ 594 anti-human CD324 (E-cadherin) antibody (clone: 67A4) and rabbit YAP polyclonal antibody, both diluted to 1:100 and 1:200, respectively. After two additional PBS washes, secondary antibodies Alexa Fluor™ 488 goat anti-rabbit IgG (H + L) and Rhodamine Red™-X goat anti-mouse IgG (H + L) were added, both diluted 1:200 and 1:250, respectively. After two additional PBS washes, DAPI staining was applied for 15 min. The samples were then subjected to a final round of PBS washes before observation under a microscope (EVOS M5000, Invitrogen). The relative red intensity of the resulting fluorescence images was quantified using ImageJ software (version 1.52a) based on grayscale values.

### 4.6. Statistical Analyses

All data are expressed as mean ± standard deviation (SD). Each experiment was repeated three times unless otherwise indicated. Statistical evaluation was performed using one-way ANOVA. A value of *p* < 0.05 was considered statistically significant. Statistical analysis was performed using the statistical software GraphPad Prism 9.

## Figures and Tables

**Table 1:** Spheroid migration distance and area on days 1, 4, and 7. The symbols * indicate that cells spread out of the microscope images. Data are based on triplicate measurements (n = 3).

Substrates	Time (Day)	Migration Distance (μm)	Migration Area (Pixels)
Non-coated	1	626 ± 92	2205 ± 203
	4	1757 ± 116	13,489 ± 544
	7	2621 ± 49	* 37,890 ± 995
Collagen-coated	1	802 ± 59	2908 ± 166
	4	2327 ± 82	21,962 ± 1046
	7	3600 ± 614	* 41,521 ± 949
Collagen hydrogel	1	1063 ± 93	4710 ± 168
	4	2588 ± 107	29,719 ± 1491
	7	4024 ± 117	* 42,903 ± 0

## Data Availability

The data presented in this study are openly available in article.

## Supplementary Materials

The following supporting information can be downloaded at: https://www.mdpi.com/article/10.3390/gels9120922/s1, Figure S1: ATR FT-IR of plastic (black), collagen (blue) and collagen with riboflavin 5′-phosphate (orange). The peaks of each functional group: Amide A: 3309 cm^−1^, Amide B: 2930 cm^−1^, Amides I: 1640 cm^−1^, Amides II: 1560 cm^−1^, Amides III: 1240 cm^−1^, C-O: 1170 cm^−1^.

## References

[1] Ross J.M., Patrick C.W., Mikos A.G., McIntire L.V., Langer R.S. (1998). Chapter II. 1—Cell-Extracellular Matrix Interactions. Frontiers in Tissue Engineering.

[2] Muntz I., Fenu M., van Osch G.J.V.M., Koenderink G.H. (2022). The role of cell–matrix interactions in connective tissue mechanics. Phys. Biol..

[3] Yi B., Xu Q., Liu W. (2022). An overview of substrate stiffness guided cellular response and its applications in tissue regeneration. Bioact. Mater..

[4] Verma B.K., Chatterjee A., Kondaiah P., Gundiah N. (2023). Substrate Stiffness Modulates TGF-&beta; Activation and ECM-Associated Gene Expression in Fibroblasts. Bioengineering.

[5] Jawad H., Brown R.A., Moo-Young M. (2011). 5.05—Mesoscale Engineering of Collagen as a Functional Biomaterial. Comprehensive Biotechnology.

[6] Elango J., Hou C., Bao B., Wang S., Maté Sánchez de Val J.E., Wenhui W. (2022). The Molecular Interaction of Collagen with Cell Receptors for Biological Function. Polymers.

[7] Shao J., Weng L., Li J., Lin H., Wang H., Lin J. (2022). Regulation of Macrophage Polarization by Mineralized Collagen Coating to Accelerate the Osteogenic Differentiation of Mesenchymal Stem Cells. ACS Biomater. Sci. Eng..

[8] Castro-Abril H., Heras J., del Barrio J., Paz L., Alcaine C., Aliácar M.P., Garzón-Alvarado D., Doblaré M., Ochoa I. (2023). The Role of Mechanical Properties and Structure of Type I Collagen Hydrogels on Colorectal Cancer Cell Migration. Macromol. Biosci..

[9] Kang Y., Kim J.H., Kim S.Y., Koh W.-G., Lee H.J. (2021). Blue Light-Activated Riboflavin Phosphate Promotes Collagen Crosslinking to Modify the Properties of Connective Tissues. Materials.

[10] Zhang Y., Wang Y., Li Y., Yang Y., Jin M., Lin X., Zhuang Z., Guo K., Zhang T., Tan W. (2023). Application of Collagen-Based Hydrogel in Skin Wound Healing. Gels.

[11] D’Urso M., Kurniawan N.A. (2020). Mechanical and Physical Regulation of Fibroblast–Myofibroblast Transition: From Cellular Mechanoresponse to Tissue Pathology. Front. Bioeng. Biotechnol..

[12] Fang Y., Eglen R.M. (2017). Three-Dimensional Cell Cultures in Drug Discovery and Development. SLAS Discov..

[13] Cao H., Duan L., Zhang Y., Cao J., Zhang K. (2021). Current hydrogel advances in physicochemical and biological response-driven biomedical application diversity. Signal Transduct. Target. Ther..

[14] Ponsonnet L., Comte V., Othmane A., Lagneau C., Charbonnier M., Lissac M., Jaffrezic N. (2002). Effect of surface topography and chemistry on adhesion, orientation and growth of fibroblasts on nickel–titanium substrates. Mater. Sci. Eng. C.

[15] Cai S., Wu C., Yang W., Liang W., Yu H., Liu L. (2020). Recent advance in surface modification for regulating cell adhesion and behaviors. Nanotechnol. Rev..

[16] Al-Azzam N., Alazzam A. (2022). Micropatterning of cells via adjusting surface wettability using plasma treatment and graphene oxide deposition. PLoS ONE.

[17] Kanta J. (2015). Collagen matrix as a tool in studying fibroblastic cell behavior. Cell Adhes. Migr..

[18] Wang Q., Chi L. (2022). The Alterations and Roles of Glycosaminoglycans in Human Diseases. Polymers.

[19] Silva J.C., Carvalho M.S., Han X., Xia K., Mikael P.E., Cabral J.M.S., Ferreira F.C., Linhardt R.J. (2019). Compositional and structural analysis of glycosaminoglycans in cell-derived extracellular matrices. Glycoconj. J..

[20] Amirrah I.N., Lokanathan Y., Zulkiflee I., Wee M.F.M.R., Motta A., Fauzi M.B. (2022). A Comprehensive Review on Collagen Type I Development of Biomaterials for Tissue Engineering: From Biosynthesis to Bioscaffold. Biomedicines.

[21] Van Zundert I., Fortuni B., Rocha S. (2020). From 2D to 3D Cancer Cell Models—The Enigmas of Drug Delivery Research. Nanomaterials.

[22] Tang D.D., Gerlach B.D. (2017). The roles and regulation of the actin cytoskeleton, intermediate filaments and microtubules in smooth muscle cell migration. Respir. Res..

[23] Zhang Q., Yu Y., Zhao H. (2016). The effect of matrix stiffness on biomechanical properties of chondrocytes. Acta Biochim. Biophys. Sin..

[24] Childers R.C., Lucchesi P.A., Gooch K.J. (2021). Decreased Substrate Stiffness Promotes a Hypofibrotic Phenotype in Cardiac Fibroblasts. Int. J. Mol. Sci..

[25] Totaro A., Panciera T., Piccolo S. (2018). YAP/TAZ upstream signals and downstream responses. Nat. Cell Biol..

[26] Li Y., Wang J., Zhong W. (2021). Regulation and mechanism of YAP/TAZ in the mechanical microenvironment of stem cells (Review). Mol. Med. Rep..

[27] Mendonsa A.M., Na T.-Y., Gumbiner B.M. (2018). E-cadherin in contact inhibition and cancer. Oncogene.

[28] Chu Y.-S., Thomas W.A., Eder O., Pincet F., Perez E., Thiery J.P., Dufour S. (2004). Force measurements in E-cadherin–mediated cell doublets reveal rapid adhesion strengthened by actin cytoskeleton remodeling through Rac and Cdc42. J. Cell Biol..

[29] O’Connor J.W., Mistry K., Detweiler D., Wang C., Gomez E.W. (2016). Cell-cell contact and matrix adhesion promote αSMA expression during TGFβ1-induced epithelial-myofibroblast transition via Notch and MRTF-A. Sci. Rep..

[30] Loh C.-Y., Chai J.Y., Tang T.F., Wong W.F., Sethi G., Shanmugam M.K., Chong P.P., Looi C.Y. (2019). The E-Cadherin and N-Cadherin Switch in Epithelial-to-Mesenchymal Transition: Signaling, Therapeutic Implications, and Challenges. Cells.

