# Supplementary material for: Investigating the Impact of Mechanical Properties and Cell-Collagen Interaction on NIH3T3 Function: A Comparative Study on Different Substrates and Culture Environments
Source: Gels. 2023 Nov 22;9(12):922. doi: 10.3390/gels9120922 (PMC10742811; doi:10.3390/gels9120922)
Supplement: Supplementary file 1 [file gels-09-00922-s001.zip › gels-2717083-supplementary.pdf]

Supplementary Materials

Investigating the Impact of Mechanical Properties and Cell-Collagen Interaction on NIH3T3 Function: A Comparative Study on Different Substrates and Culture Environments

A Yeon Cho [1] and Hyun Jong Lee [1,*]

[1] Department of Chemical and Biological Engineering, Gachon University, 1342 Seongnam-daero, Seongnam-si 13120, Republic of Korea

* Correspondence: hjlee2@gachon.ac.kr (H.J.L.)

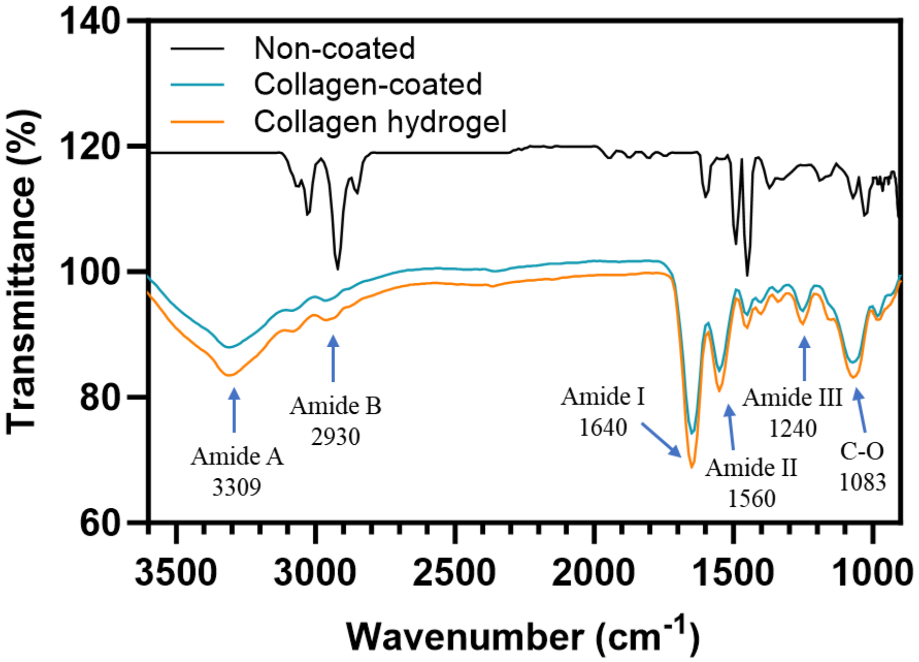

Figure S1. ATR FT-IR of plastic (black), collagen (blue) and collagen with riboflavin 5'-phosphate (orange). The peaks of each functional group: Amide A: 3309 cm-1, Amide B: 2930 cm-1, Amides I: 1640 cm-1, Amides II: 1560 cm-1, Amides III: 1240 cm-1, C-O: 1170 cm-1.
